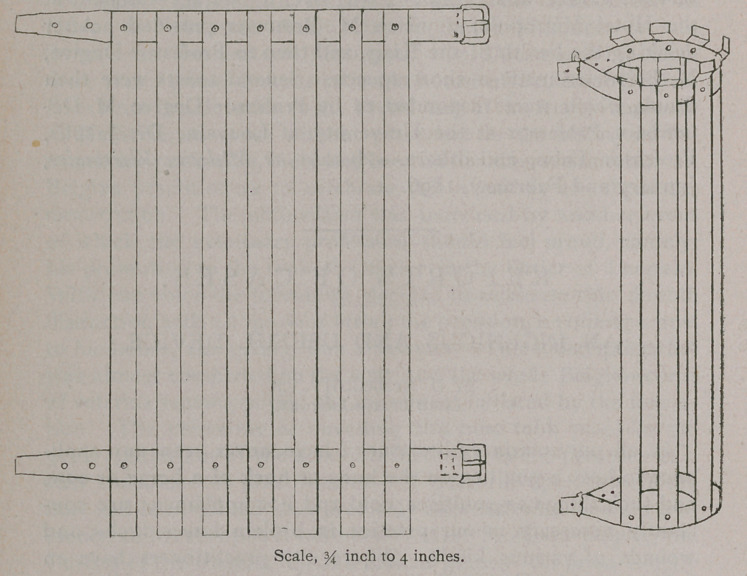# An Ingenious and Useful Device

**Published:** 1896-06

**Authors:** D. McNaught

**Affiliations:** Rapid City, Manitoba


### REPORTS OF CASES.

### AN INGENIOUS AND USEFUL DEVICE.

By D. McNAUGHT, V.S.,
RAPID CITY, MANITOBA.

In all our veterinary literature I have never seen any appliance to keep a poultice on the knee or hock of a horse or cow, and such things as poultices, cold and hot appliances, are constantly necessary in our practice, in broken knees, kicks, and wounds of various kinds. Where city practitioners have an infirmary with all the appliances for the continued application of hot and cold water, etc., many things can be done. But most of the actual practice is done in farmers' stables, where no appliances are at hand. There a man must be shifty and suit himself to his surroundings.

A very bad case of broken knees brought out this simple little invention, and I now give it to the profession for what it is worth. It is both simple and cheap, and can be made by any veterinarian, and there is no patent on it.

It consists of two straps and seven pieces of hoop-iron as light as will sustain the weight of the poultice. The straps are ten inches long and three-quarters of an inch wide, of good stout leather, with a buckle on one end. Lay down the straps fourteen inches apart, or whatever length is required for the animal under treatment; cut off seven pieces of light hoop-iron threequarters of an inch wide, with two holes in each, as represented in cut, rivet on with short copper rivets, keeping the head inside on the leather, then in a vise, or any other way handy, turn over one inch. Then turn the last inch up, as shown in cut, having previously rounded the corners so as not to catch or

Scale, ¾ inch to 4 inches.

tear the application. Take a soft rag and wind around the lower strap so as to make it rest easy on the foot; also a small quantity around the top so that it will adhere more closely to the leg. This appliance can be made by any handy man at a cost of not more than fifty cents, and will last for many years by keeping the band-iron well oiled.

## Figures and Tables

**Figure f1:**